# Differential Path-Length Factor's Effect on the Characterization of Brain's Hemodynamic Response Function: A Functional Near-Infrared Study

**DOI:** 10.3389/fninf.2018.00037

**Published:** 2018-06-20

**Authors:** Muhammad A. Kamran, Malik M. N. Mannann, Myung Yung Jeong

**Affiliations:** Department of Cogno-Mechatronics Engineering, Pusan National University, Busan, South Korea

**Keywords:** functional near-infrared spectroscopy, differential path-length factor, hemodynamic response, optimal cortical model, optical brain imaging

## Abstract

Functional near-infrared spectroscopy (fNIRS) has evolved as a neuro-imaging modality over the course of the past two decades. The removal of superfluous information accompanying the optical signal, however, remains a challenge. A comprehensive analysis of each step is necessary to ensure the extraction of actual information from measured fNIRS waveforms. A slight change in shape could alter the features required for fNIRS-BCI applications. In the present study, the effect of the differential path-length factor (DPF) values on the characteristics of the hemodynamic response function (HRF) was investigated. Results were compiled for both simulated data sets and healthy human subjects over a range of DPF values from three to eight. Different sets of activation durations and stimuli were used to generate the simulated signals for further analysis. These signals were split into optical densities under a constrained environment utilizing known values of DPF. Later, different values of DPF were used to analyze the variations of actual HRF. The results, as summarized into four categories, suggest that the DPF can change the main and post-stimuli responses in addition to other interferences. Six healthy subjects participated in this study. Their observed optical brain time-series were fed into an iterative optimization problem in order to estimate the best possible fit of HRF and physiological noises present in the measured signals with free parameters. A series of solutions was derived for different values of DPF in order to analyze the variations of HRF. It was observed that DPF change is responsible for HRF creep from actual values as well as changes in HRF characteristics.

## Introduction

Optical spectroscopy is an emerging neuro-imaging modality that can indicate cortical functionality with good temporal resolution relative to the other modalities (e.g., Mannan et al., 2016a; Meszlényi et al., 2017). The optical signal observed through functional near-infrared spectroscopy (fNIRS) reflects the interaction of light with matter, and will differ according to the properties of matter (Maikala, 2010). fNIRS utilizes near-infrared (NIR) light in the spectral range of 650–900 nm. Thus, absorption and scattering are among the fundamental characteristics/properties that produce signal/NIR light attenuation (van der Zee et al., 1990; Kohl et al., 1998). It is well-known that biological tissue is a medium that highly scatters NIR light (Duncan et al., 1995). The absorption of NIR light is mainly caused by two chromophores in the blood, namely, oxy-hemoglobin (HbO) and deoxy-hemoglobin (HbR) (Schroeter et al., 2004). Continuous wave near-infrared spectroscopy (CW-NIRS) measures the concentration changes of HbO (Δ*Hbo*) and HbR (Δ*HbR*), respectively, at each time step, making it at attractive option for brain-computer interface (BCI) applications, among others (Scholkmann and Wolf, 2013; Talukdar et al., 2013; Naseer and Hong, 2015; Mannan et al., 2016b; Cavazza et al., 2017; García-Prieto et al., 2017).

The fNIRS system consists mainly of a source-emitting NIR light of dual or more wavelengths of particular frequencies, along with detector(s) to measure the intensity of attenuated light. NIR light of multiple wavelengths (e.g., 760 and 830 nm) is thrown onto the surface of the scalp, with a physical connection between the source and scalp. This incident NIR light passes through the scalp, skull, cerebral spinal fluid (CSF), gray matter and white matter, a portion of it being received at a nearby photo-detector. It is assumed that the NIR light emitted by the source and detected by a near-by detector follows a banana-shaped path (Kamran et al., 2016). Thus, the actual path traveled by NIR light is much longer than the source-detector separation on the surface of the scalp (Delpy et al., 1988; Hiraoka et al., 1993; Chatterjee et al., 2015; Piao et al., 2015).

It is necessary to accurately estimate/measure the actual distance traveled by NIR light (Kohl et al., 1998). A parameter, namely the differential path-length factor (DPF), has been used in previous studies to cover the extra distance traveled by NIR light (Kamran et al., 2016). This is a wavelength-dependent scaling factor that indicates how many times farther (than the actual source/detector separation) the detected NIR light has traveled (Scholkmann and Wolf, 2013). It has been common practice to use a DPF value between three and six (Delpy et al., 1988; Duncan et al., 1995; Scholkmann and Wolf, 2013). The value of DPF is generally determined through measurement of the mean time of flight (TOF) of a pico-second pulse of NIR light traveling through biological tissue (van der Zee et al., 1990, 1992; Ferrari et al., 1992). Another possibility for DPF measurement is via frequency-domain NIRS (FD-fNIRS) (Lackowicz and Berndt, 1990; Duncan et al., 1993, 1995). Duncan et al. (1995) determined the values of DPF at four different wavelengths (i.e., 690, 744, 807, 832 nm) for adult arm, head, leg, and new-born-baby head, respectively. Later, Duncan et al. (1996) experimented with 283 subjects (age range: 1 day ~ 50 years) and developed four different equations for the age dependency of DPF at particular wavelengths. The equations are as follows:

(1) $$\begin{array}{rcl} DPF^{690} & = & 5.38+0.049^{*}\left(A^{0.877}\right), \end{array}$$

(2) $$\begin{array}{rcl} DPF^{744} & = & 5.11+0.106^{*}\left(A^{0.723}\right), \end{array}$$

(3) $$\begin{array}{rcl} DPF^{807} & = & 4.99+0.067^{*}\left(A^{0.814}\right), \end{array}$$

(4) $$\begin{array}{rcl} DPF^{832} & = & 4.67+0.062^{*}\left(A^{0.819}\right), \end{array}$$

Later, Kohl et al. (1998) concluded that the DPF is the ratio of the attenuation change to the change in absorption coefficient, which is to say, ($DPF\;=\;\frac{\partial A}{\partial \mu_{a}}r^{-1}$), where A is the attenuation, [μ_*a*_] is the absorption coefficient, and *r* is the source-detector separation. Schroeter et al. (2003) analyzed the age dependency of the hemodynamic response function on the DPF. Their results suggest that aging decreases the hemodynamic response and that the decrease might be caused by the changing of tissue properties with age. In their subsequent work, Schroeter et al. (2004) pointed out that the DPF can vary intra-individually between several pixels and inter-individually between subjects of the same age. Their observation was based on previous studies (Essenpreis et al., 1993; Zhao et al., 2002). Since none of the commercially available optical imaging systems measures DPF for each pixel, such studies are impossible (Schroeter et al., 2004). The multiple layers of the human scalp and skull can affect the DPF, thereby leading to systematic errors (Talukdar et al., 2013). Recently, Scholkmann and Wolf (2013) modeled the DPF as a function of age and wavelength, deriving the following equation (Equation 5) for calculation of DPF at any wavelength and age A:

$$\begin{array}{rcl} DPF\left(\lambda ,A\right) & = & 223.3+0.05624A^{0.8493}-5.723^{*}10^{-7}\lambda^{3} \end{array}$$

(5) $$\begin{array}{r} +0.001245\lambda^{2}-0.9025\lambda . \end{array}$$

It is obvious from the previous studies that the DPF can affect optically measured signals and incur, thereby, error and inaccurate results (Jasdzewski et al., 2003). Therefore, it is very important to analyze the effects of the DPF on NIRS waveforms. In the current study, the authors analyzed the effect of DPF on optically measured signals. In the first step, simulated data sets were generated using a method described in previous studies (Prince et al., 2003; Kamran et al., 2015). The simulated HbO signal was separated into its constituent optical densities under different constraints, and the accuracy of the results were confirmed before further processing. The results were observed for variations of ${\text{DPF}}^{\lambda_{1}}$ and ${\text{DPF}}^{\lambda_{2}}$ and compared with the actual signals. Later, NIRS experiments were performed on six healthy subjects. The problem was formulated as an iterative optimization problem for estimation of the best possible fit of HRF and physiological noises with free parameters. Comparative results were derived, and a detailed analysis was performed for different values of ${\text{DPF}}^{\lambda_{1}}$ and ${\text{DPF}}^{\lambda_{2}}$. The results serve to comprehensively summarize the effect of DPF variations on HRF.

## Theory

### Derivation of concentration changes of chromophores

The intensity of attenuated NIR light received by a detector depends on the reflection, absorption and scattering properties of the tissues that light has passed through (Delpy et al., 1988; Duncan et al., 1996; Maikala, 2010; Kamran et al., 2016). This phenomenon can be modeled according to the modified beer-Lambert law (MBLL) as

(6) $$\begin{array}{r} OD=\ln\;\left(\frac{I_{in}\left(\lambda \right)}{I_{o}\left(\lambda \right)}\right) \end{array}$$

Or

(7) $$\begin{array}{r} OD=\Delta \mu_{a}\left(\lambda \right)dDPF\left(\lambda \right)+G\left(\lambda \right) \end{array}$$

where *I*_*in*_(λ) and *I*_0_(λ) are the incident and attenuated detected light, respectively, μ_*a*_ is the absorption coefficient, *d* is the source-detector separation, and *G*(λ) is the geometrical parameter for the light's scattering properties. The absorption coefficient is the sum of the products of the extinction coefficients and the concentrations of the different chromophores present in the medium under experimentation (Scholkmann and Wolf, 2013; Kamran et al., 2016). The above equation can be modified, by scattering to be considered as a constant (Maikala, 2010). Finally, we obtain

(8) $$\begin{array}{r} \Delta OD^{\lambda_{i}}=\left(\varepsilon_{HbO}^{\lambda_{i}}\Delta HbO+\varepsilon_{HbR}^{\lambda_{i}}\Delta HbR\right)dDPF\left(\lambda \right) \end{array}$$

where [λ_*i*_] is the wavelength of incident NIR light, and $\varepsilon_{HbO}^{\lambda_{i}}$ and $\varepsilon_{HbR}^{\lambda_{i}}$ are the extinction coefficients of HbO and HbR, respectively. Assuming two wavelengths λ_1_ and λ_2_, we can rewrite the above equation as

(9) $$\begin{array}{r} \Delta HbO^{i}\left(k\right)=\frac{\left(\varepsilon_{HbR}^{\lambda_{1}}\frac{\Delta OD^{\lambda_{2}}\left(k\right)}{DPF^{\lambda_{2}}}\right)-\left(\varepsilon_{HbR}^{\lambda 2}\frac{\Delta OD^{\lambda_{1}}\left(k\right)}{DPF^{\lambda_{1}}}\right)}{l^{i}\left(\varepsilon_{HbR}^{\lambda_{1}}\varepsilon_{HbO}^{\lambda_{2}}-\varepsilon_{HbR}^{\lambda_{2}}\varepsilon_{HbO}^{\lambda_{1}}\right)} \end{array}$$

and

(10) $$\begin{array}{r} \Delta HbR^{i}\left(k\right)=\frac{\left(\varepsilon_{HbO}^{\lambda_{2}}\frac{\Delta OD^{\lambda_{1}}\left(k\right)}{DPF^{\lambda_{1}}}\right)-\left(\varepsilon_{HbO}^{\lambda_{1}}\frac{\Delta OD^{\lambda_{2}}\left(k\right)}{DPF^{\lambda_{2}}}\right)}{l^{i}\left(\varepsilon_{HbR}^{\lambda_{1}}\varepsilon_{HbO}^{\lambda_{2}}-\varepsilon_{HbR}^{\lambda_{2}}\varepsilon_{HbO}^{\lambda_{1}}\right)} \end{array}$$

where Δ*HbO*^*i*^(*k*) and Δ*HbR*^*i*^(*k*) are the relative concentration changes of HbO and HbR, respectively, *k* is the step time, *i* represents the *i*th-channel of the source-detector set, λ_1_ and λ_2_ are NIR light of 760 and 830 nm wavelength, respectively, $\varepsilon_{HbO}^{\lambda_{1}}$, $\varepsilon_{HbR}^{\lambda_{1}}$, $\varepsilon_{HbO}^{\lambda_{2}}$, and $\varepsilon_{HbR}^{\lambda_{2}}$ represent the extinction coefficients of HbO and HbR at two different wavelengths, respectively, $\Delta {OD}^{\lambda_{j}}\left(k\right)$ is the optical density variation at the *k*th-sample time and a particular wavelength (*j* = 1, 2), *l*^*i*^ is the source-detector separation, and ${DPF}^{\lambda_{j}}$ is the differential path-length factor at a particular wavelength (*j* = 1, 2).

### Hemodynamic response model

Since the fNIRS-measured cortical signal resembles one measured through functional magnetic resonance imaging (fMRI), Friston et al. (1994) introduced statistical parameter mapping (SPM) software for analysis of fMRI data. The general characteristics of the hemodynamic response (HR) for stimulus are well-established in the literature. There is early de-oxygenation at 1–3 s, followed by a positive peak at around 5–6 s, a drop-down to the baseline, and, finally, a settling down at 25–30 s (Friston et al., 1998; Koray et al., 2008). The canonical shape of this model can be represented as a linear combination of two Gamma functions (Friston et al., 1994; Kamran et al., 2015),

(11) $$\begin{array}{r} HRF\left(k\right)=h{\left(k\right)}^{*}u\left(k\right), \end{array}$$

(12) $$\begin{array}{r} h\left(k\right)=\left[\frac{k^{\alpha_{1}-1}\beta_{{}_{1}}^{\alpha_{1}}e^{-\beta_{1}k}}{\Gamma \left(\alpha_{1}\right)}-\frac{k^{\alpha_{2}-1}\beta_{{}_{2}}^{\alpha_{2}}e^{-\beta_{2}k}}{6\Gamma \left(\alpha_{2}\right)}\right] \end{array}$$

where *u* he experimental procedure, *h* is the canonical hemodynamic response function (cHRF), α_1_ is the delay of the response, α_2_ is the delay of the undershoot, β_1_ is the dispersion of the response, β_2_ is the dispersion of the undershoot, and Γ represents the Gamma distribution.

### Optical signal model

In the case of fNIRS, the observed processed signal is a linear combination of cortical activity relevant to a particular experiment, physiological signals and certain unknown signals termed as “Gaussain noise.” The signal related to cortical activity is modeled in Equation (11). Physiological noises are respiratory rhythm, heartbeat, and low-frequency Mayer waves (Prince et al., 2003; Abdelnour and Huppert, 2009; Kamran et al., 2015). These physiological noises are periodic in nature for healthy human beings. Therefore, their corresponding signals can be added as sinusoidal signals (Prince et al., 2003; Kamran et al., 2015). Accordingly, the optical signal can be modeled mathematically as

$$\begin{array}{rcl} y_{{}_{HbO}}^{i}\left(k\right) & = & a_{o}+a_{1}HRF\left(k\right)+a_{c}\sin\left(2\pi f_{c}k\right)+a_{r}\sin\left(2\pi f_{r}k\right) \end{array}$$

(13) $$\begin{array}{r} +a_{m}\sin\left(2\pi f_{m}k\right)+\varepsilon^{i}\left(k\right) \end{array}$$

where $y_{HbO}^{i}$ is the observed HbO time-series at the *i*th-channel, a_0_ is the baseline correction, a_1_ is the activity-strength parameter, a_*c*_, a_*r*_, a_*m*_, f_*c*_, f_*r*_, f_*m*_ are the amplitudes and frequencies of the cardiac, respiratory and Mayer waves, and ε^*i*^(*k*) is the zero mean Gaussian noise at the *k*th-sample time.

### Experimental procedure and paradigm

Six healthy subjects participated in the experiments. Their average age was 28 years with a standard deviation of 7 years. Each subject was asked about his/her medical history, and it was determined that none had had any neuronal disorder. All of the subjects had 6 × 6 eyesight with/without glasses. A written consent attesting to his willingness to participate in the experiment was signed by each subject. The experiment was conducted in accordance with the latest version of the Declaration of Helsinki. The study was approved by ethical Institutional Review Board, Pusan National University, South Korea. As it is standard good practice, all of the subjects were informed of the experimental details for the best possible results. They were advised to remain calm while sitting on a comfortable seat, so as to avoid artifacts and noise. The experiment included an initial rest period of 15 s followed by four task/rest trials. Each trial included a 15–s task session followed by a 15–s rest session. During the task sessions, different arithmetic operations with answers were shown; the subject was instructed to tap his right index finger if the answer was correct, and to keep the finger still if the answer was incorrect. It was observed that all of the subjects properly tapped their finger only on display of a correct answer. The arithmetic operations were shown on a screen positioned 100 cm away from the subject. The data were acquired at a sampling rate of 1.81 Hz. Later, it was resampled at a rate of 100 Hz for further processing.

The algorithm was verified on simulated data sets of different combinations of stimulus patterns and task durations (10, 20, and 30 s). The simulated data sets were generated using Equations (11–13) according to the method described in Kamran et al. (2015).

### Problem formulation

There are three commercially available NIRS systems: time domain (TD), frequency domain (FD), and continuous wave (CW) (Talukdar et al., 2013). In continuous-wave NIRS (CW-NIRS), usually, two different wavelengths of NIR light are thrown onto the scalp, and their corresponding changes in optical density are received by detector/detectors. In that way, the optical densities corresponding to wavelengths λ_1_ and λ_2_ are recorded (Ye et al., 2009). These optical densities $\Delta {OD}^{\lambda_{1}}$ and $\Delta {OD}^{\lambda_{2}}$ are used to obtain the concentration changes of HbO and HbR by Equations (9, 10). The problem with respect to simulated data sets is formulated as a cost function *j*_1_,

(14) $$\begin{array}{r} J_{1}=\overset{N}{\underset{k=1}{\sum }}{\left\{y_{HbO}^{i}\left(k\right)-0.0375\Delta OD^{\lambda_{2}}\left(k\right)+0.01644\Delta OD^{\lambda_{1}}\left(k\right)\right\}}^{2}. \end{array}$$

The Equation (14) is solved for optical densities $\Delta {OD}^{\lambda_{1}}$ and $\Delta {OD}^{\lambda_{2}}$] using an optimization algorithm to minimize *J*_1_ under certain constraints. The above equation has infinitely many solutions, which are divided into four categories. Correspondingly, four differently constrained problems are solved for optical densities: (1): $\Delta {OD}^{\lambda_{1}},\Delta {OD}^{\lambda_{2}}>0$, (2): $\Delta {OD}^{\lambda_{1}}>,\Delta {OD}^{\lambda_{2}}<0$, (3): $\Delta {OD}^{\lambda_{1}}<0,\Delta {OD}^{\lambda_{2}}>0$, and (4): $\Delta {OD}^{\lambda_{1}}<0,\Delta {OD}^{\lambda_{2}}<0$. The solutions for Equation (14) are fed into the equation

(15) $$\begin{array}{r} y_{HbO}^{i}\left(k\right)=0.2170\frac{\Delta OD^{\lambda_{2}}\left(k\right)}{DPF^{\lambda_{2}}}-0.1015\frac{\Delta OD^{\lambda_{1}}\left(k\right)}{DPF^{\lambda_{1}}}, \end{array}$$

and the results are compared with the actual data to verify the correctness of the solutions for actual values of ${DPF}^{\lambda_{1}}$ and ${DPF}^{\lambda_{2}}$. Later, different values of ${DPF}^{\lambda_{1}}$ and ${DPF}^{\lambda_{2}}$ are used to analyze the effects of these parameters on HRF attributes. In the case of real data sets, most of the previous studies have utilized the outputs of Equations (9, 10) for further processing and modeling of HRF signals (Jasdzewski et al., 2003; Abdelnour and Huppert, 2009; Kamran and Hong, 2013, 2014; Kamran et al., 2015). In the present study, a proposed alternative means of optimization for estimation of the HRF directly from given optical densities of real data sets was tested. For this purpose, a cost function *J*_2_,

$$\begin{array}{l} J_{2}=\sum_{k=1}^{N}\left\{(a_{o}+a_{1}HRF(k)+a_{c}\sin(2\pi f_{c}k)+a_{r}\sin(2\pi f_{r}k\right)\\ +a_{m}\sin(2\pi f_{m}k))-0.2170\frac{\Delta OD^{\lambda_{1}}(k)}{DPF^{\lambda_{2}}}-0.1015\frac{\Delta OD^{\lambda_{2}}(k)}{DPF^{\lambda_{1}}}{\}}^{2}, \end{array}$$

was defined, which can be formulated in an optimization problem according to the following constraints:

(16) $$\begin{array}{r} \begin{array}{c} \min J_{2}\left(\alpha_{1},\alpha_{2},\beta_{1},\beta_{2},a_{o},a_{1},a_{c},a_{m},a_{r},f_{c},f_{r},f_{m}\right)s.t\\ C_{1}:2\leq \alpha_{1}\leq 10,C_{7}:0\leq a_{c}\leq 2,\\ C_{2}:6\leq \alpha_{2}\leq 20,C_{8}:0\leq a_{r}\leq 2,\\ C_{3}:0.5\leq \beta_{1}\leq 2,C_{9}:0\leq a_{m}\leq 2,\\ C_{4}:0\leq \beta_{2}\leq 1.5,C_{10}:0.5\leq f_{c}\leq 1.5,\\ C_{5}:0\leq a_{o}\leq 20,C_{11}:0.2\leq f_{r}\leq 0.3,\\ C_{6}:0\leq a_{1}\leq 15.C_{12}:0.09\leq f_{m}\leq 0.1. \end{array} \end{array}$$

The optimal values of the free parameters ($\alpha_{1}^{*},\alpha_{2}^{*},\beta_{1}^{*},\beta_{1}^{*},\alpha_{0}^{*},\alpha_{1}^{*},\alpha_{c}^{*},\alpha_{m}^{*},\alpha_{r}^{*},f_{c}^{*},f_{r}^{*},f_{m}^{*},$) for specific values of $DPF^{\lambda_{1}}$ and $DPF^{\lambda_{2}}$ are estimated using an improved version of the simplex method [later named the Nelder-Mead simplex method (NMSM)]. The iteration of NMSM can be performed in three steps, namely, ordering, centroid, and transformation. The details on this algorithm are available in Haftka et al. (1990), Lagarias et al. (1998), Luersen and Riche (2004), and Kamran et al. (2015).

## Results

**Figure 1:**
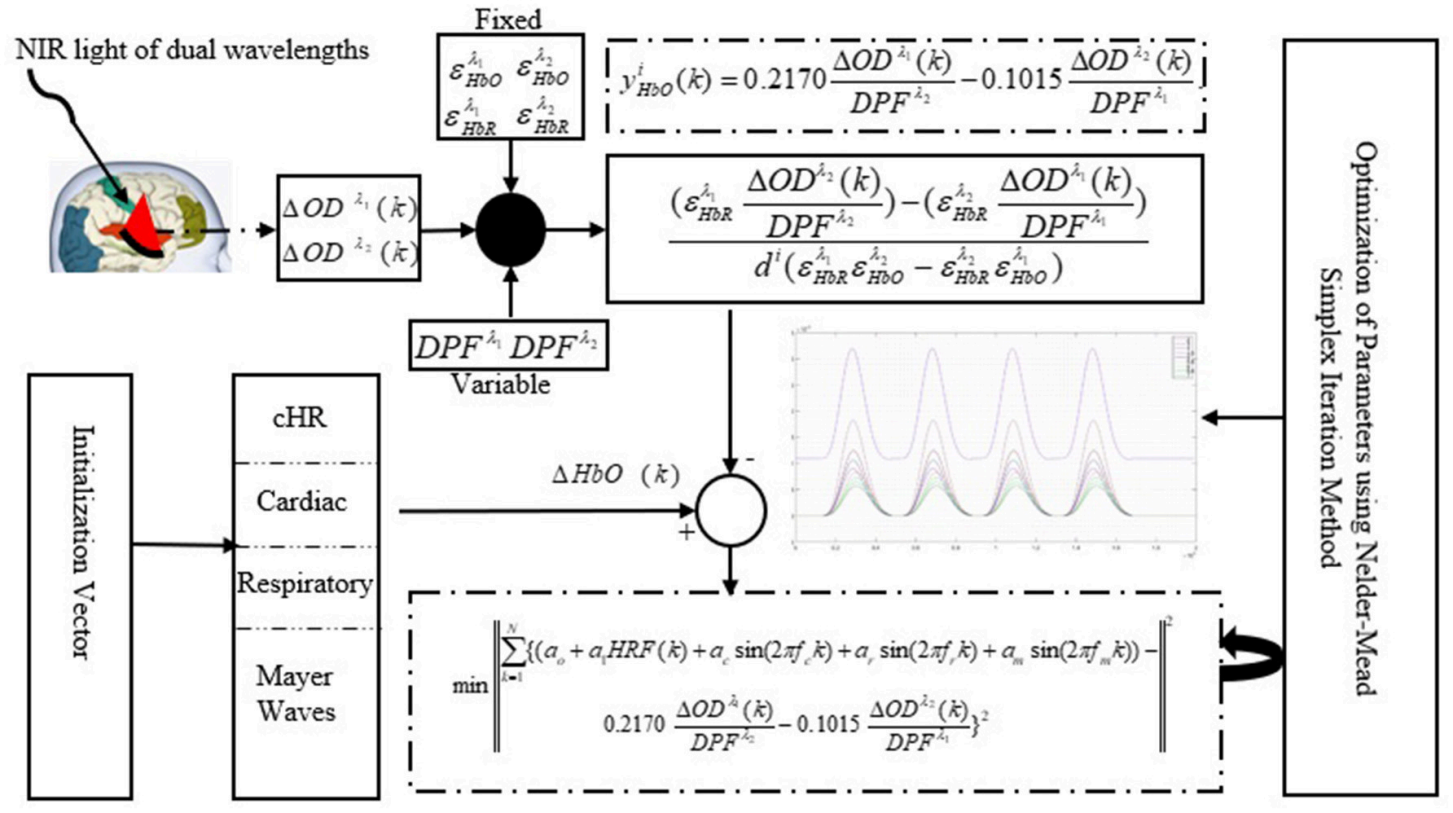
Schematic of algorithm.

**Figure 2:**
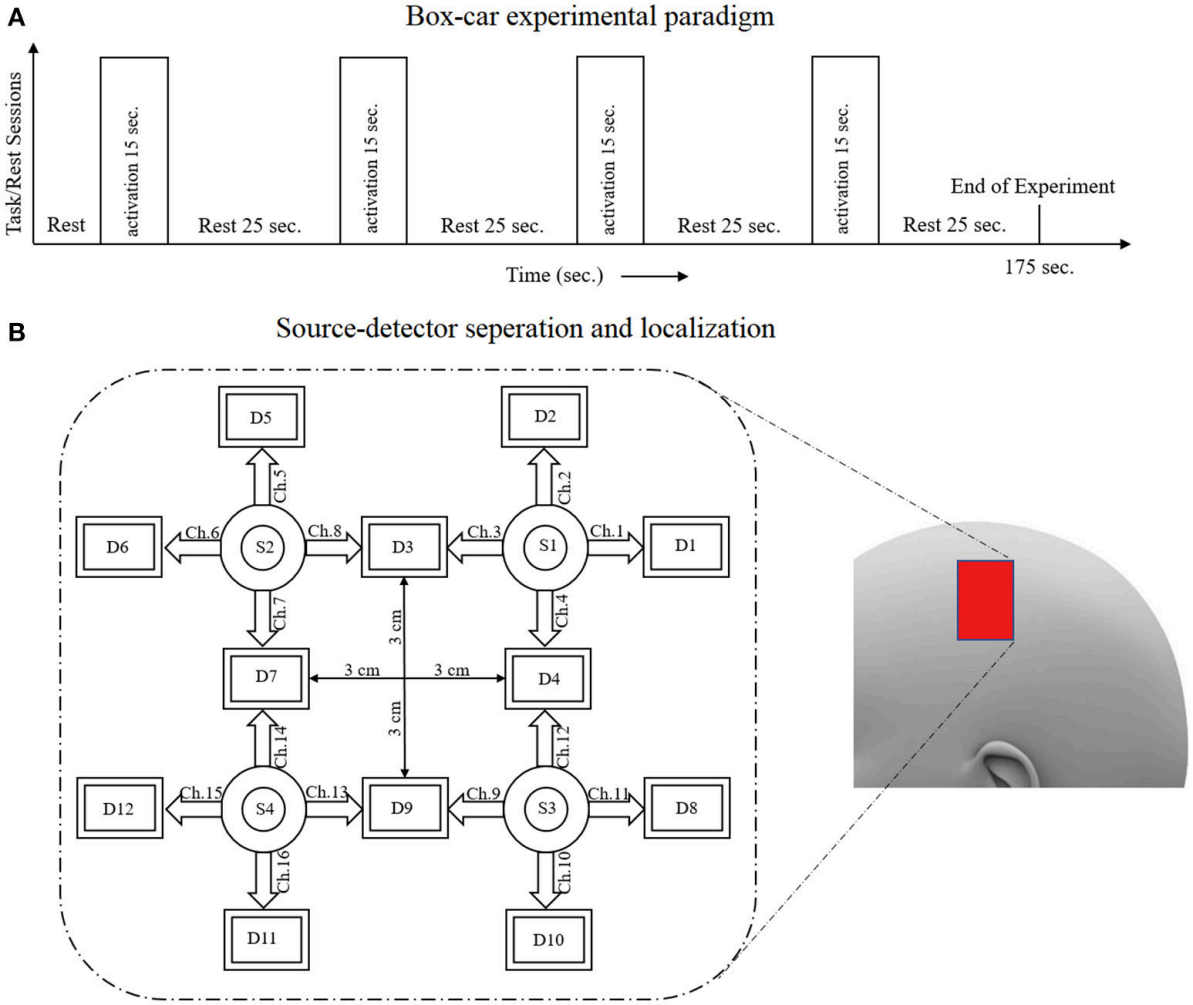
**(A)** Experimental paradigm, **(B)** source-detector localization and separation.

**Figure 3:**
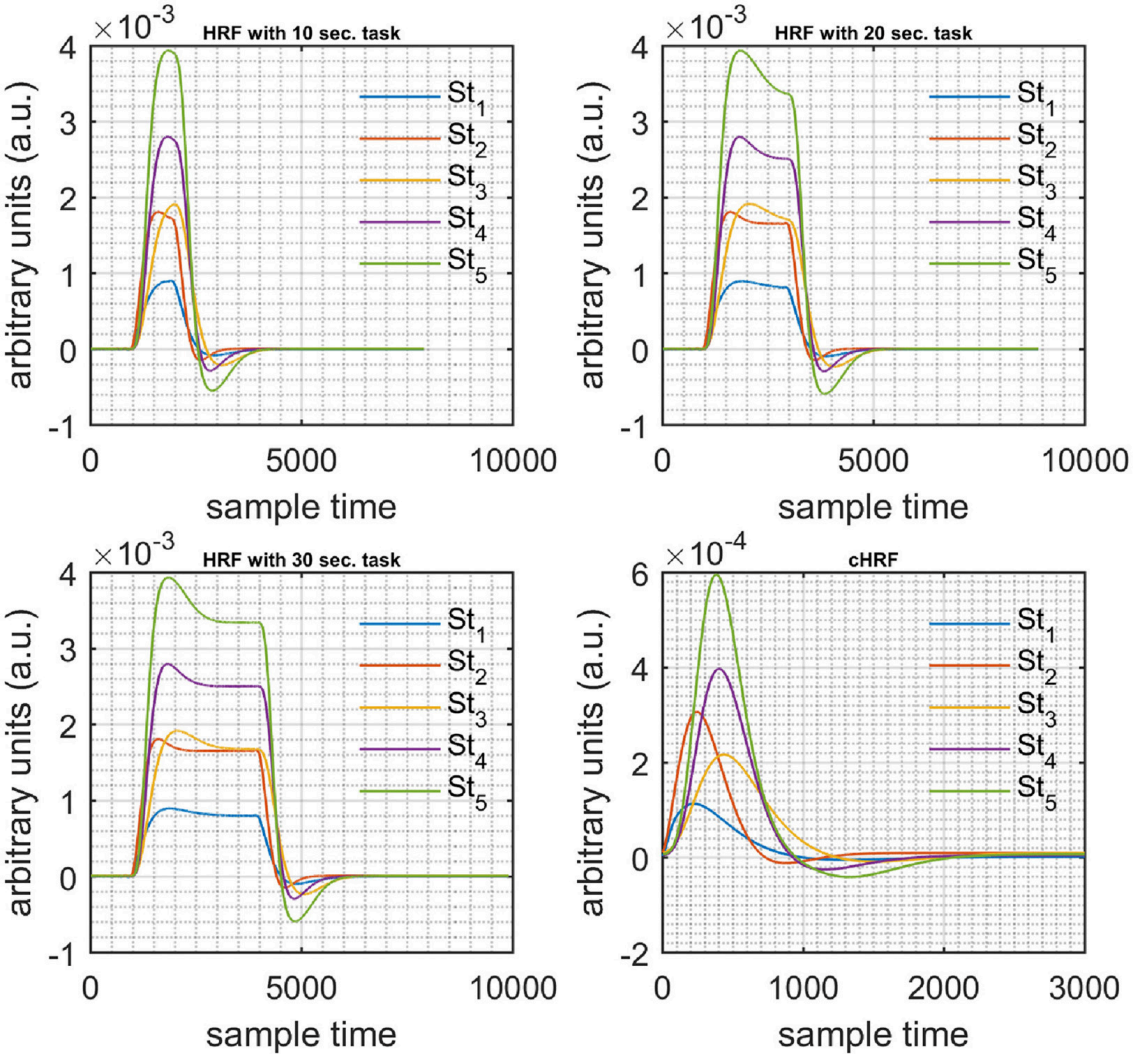
Simulated data with initial rest (10 s) different stimulus (St_1_-St_5_) and task durations (10, 20, and 30 s) followed by 30 s rest, 10 s activation (top left), 20 s activation (top right), 30 s activation (bottom left), and canonical hemodynamic response to all stimuli (bottom right).

**Figure 4:**
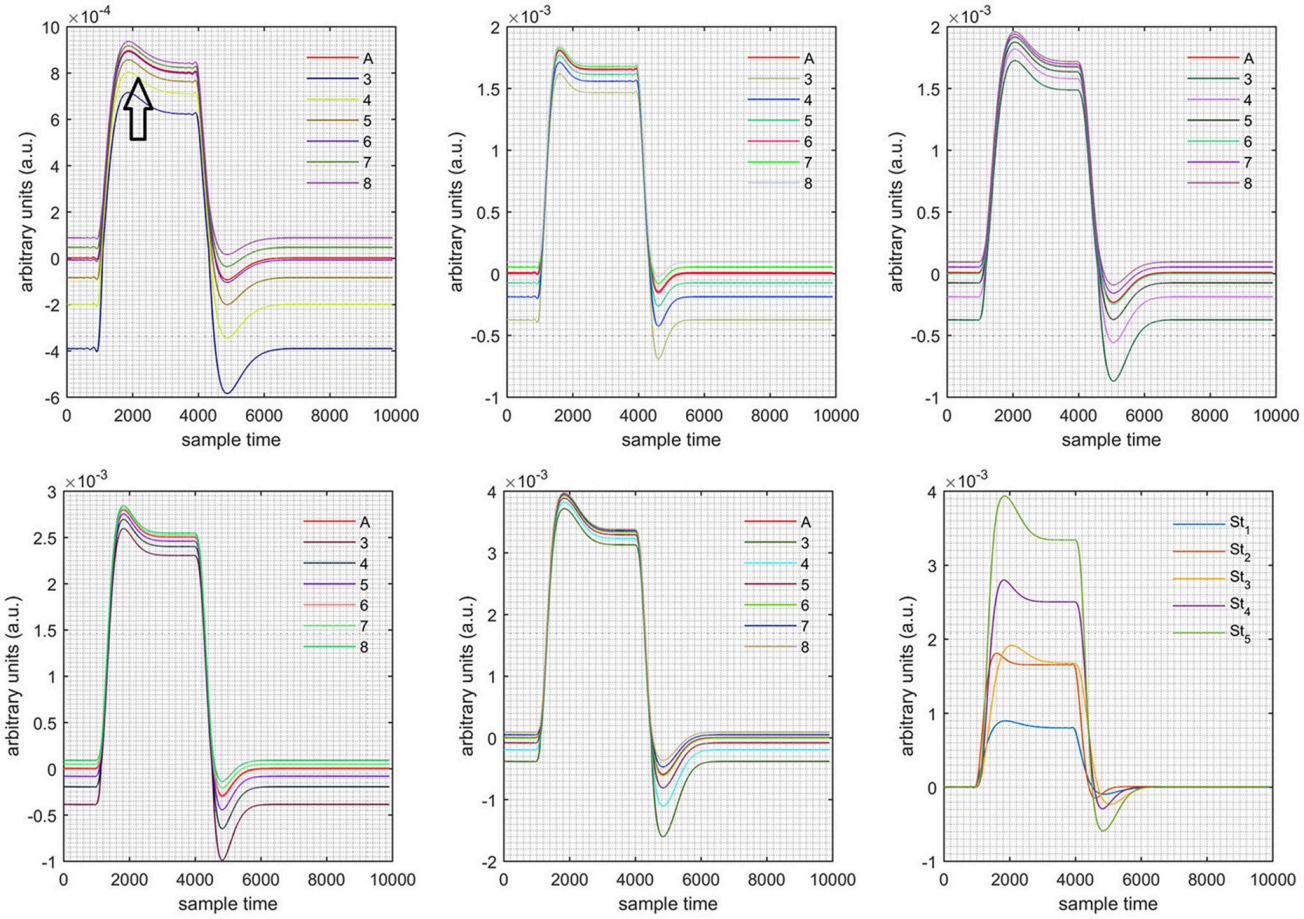
Results for both positive cases (Case: 1) with variation in value of $DPF^{\lambda_{1}}$ (3–8) under stimulation St_1_-St_5_ (top left, top middle, top right, bottom left, and bottom middle).

**Figure 5:**
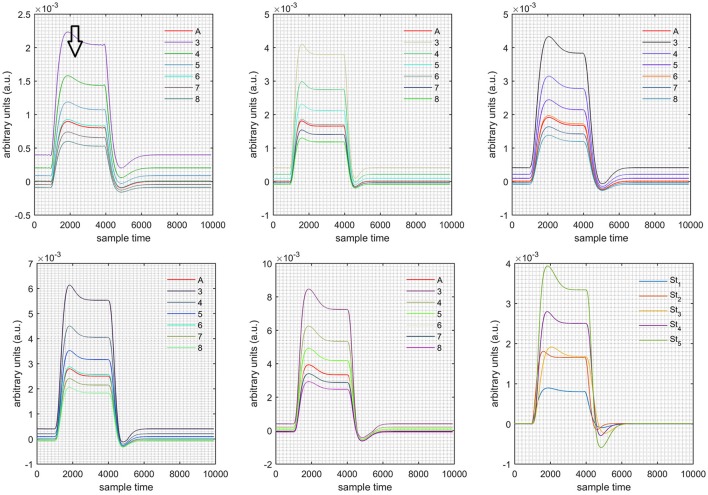
Results for both negative cases (Case: 2) with variation in value of $DPF^{\lambda_{1}}$ (3–8) under stimulation St_1_-St_5_ (top left, top middle, top right, bottom left, and bottom middle).

**Figure 6:**
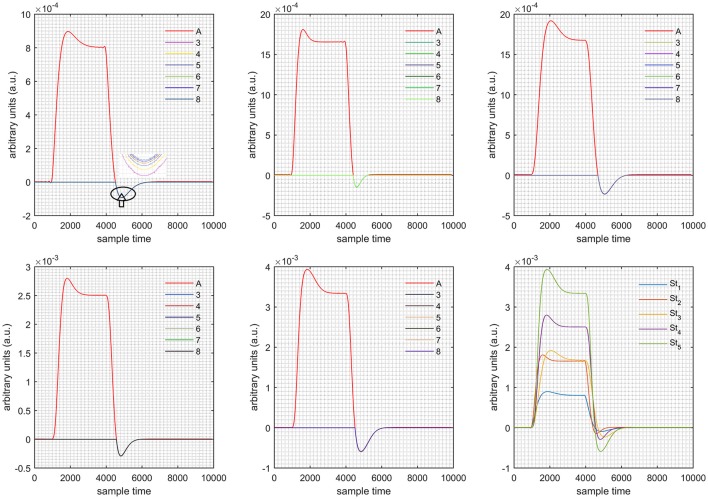
Results for first positive and second negative cases (Case: 3) with variation in value of $DPF^{\lambda_{1}}$ (3–8) under stimulation St_1_-St_5_ (top left, top middle, top right, bottom left, and bottom middle).

**Figure 7:**
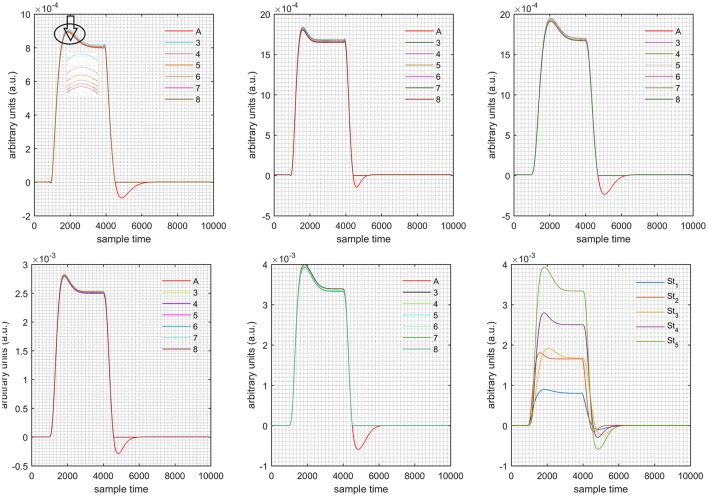
Results for first negative and second positive cases (Case: 4) with variation in value of $DPF^{\lambda_{1}}$ (3–8) under stimulation St_1_-St_5_ (top left, top middle, top right, bottom left, and bottom middle).

**Figure 8:**
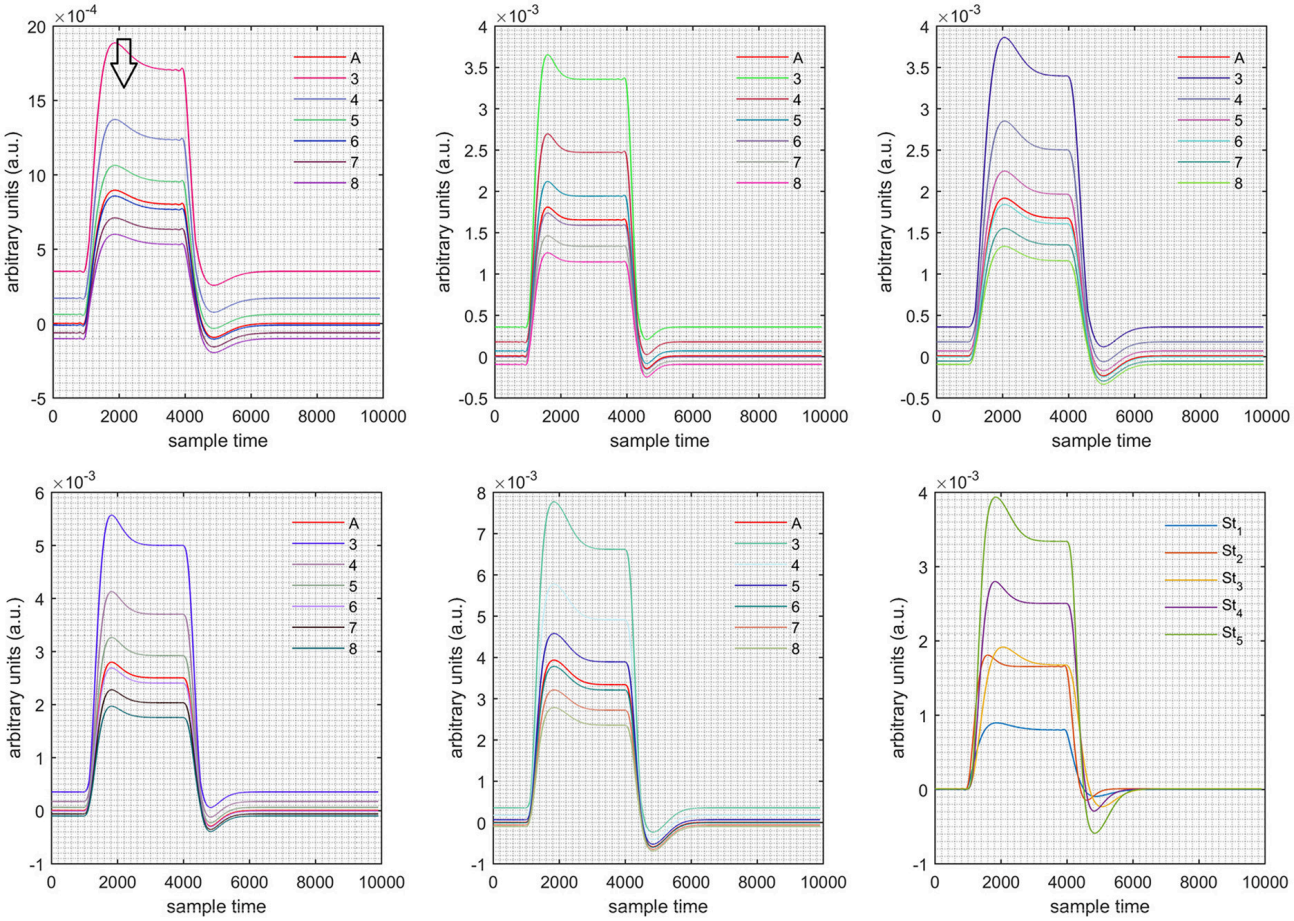
Results for both positive cases (Case: 1) with variation in value of $DPF^{\lambda_{2}}$ (3–8) under stimulation St_1_-St_5_ (top left, top middle, top right, bottom left, and bottom middle).

**Figure 9:**
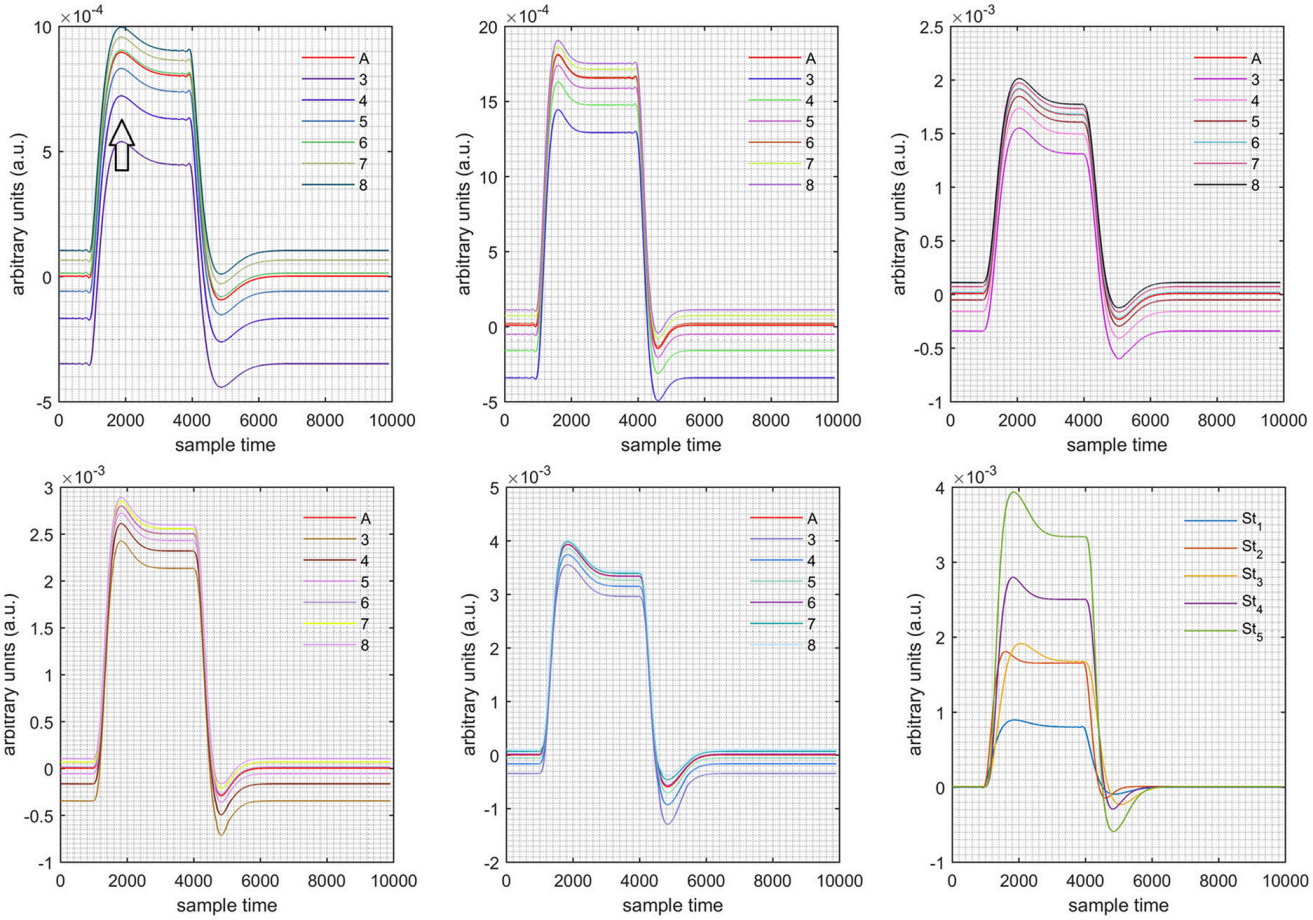
Results for both negative cases (Case: 2) with variation in value of $DPF^{\lambda_{2}}$ (3–8) under stimulation St_1_-St_5_ (top left, top middle, top right, bottom left, and bottom middle).

**Figure 10:**
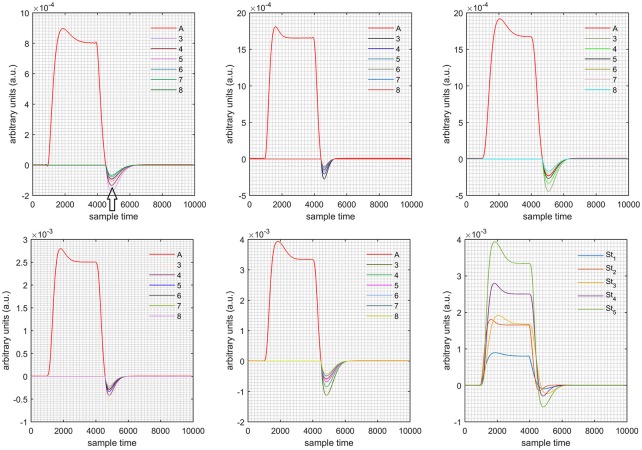
Results for first positive and second negative cases (Case: 3) with variation in value of $DPF^{\lambda_{2}}$ (3–8) under stimulation St_1_-St_5_ (top left, top middle, top right, bottom left, and bottom middle).

**Figure 11:**
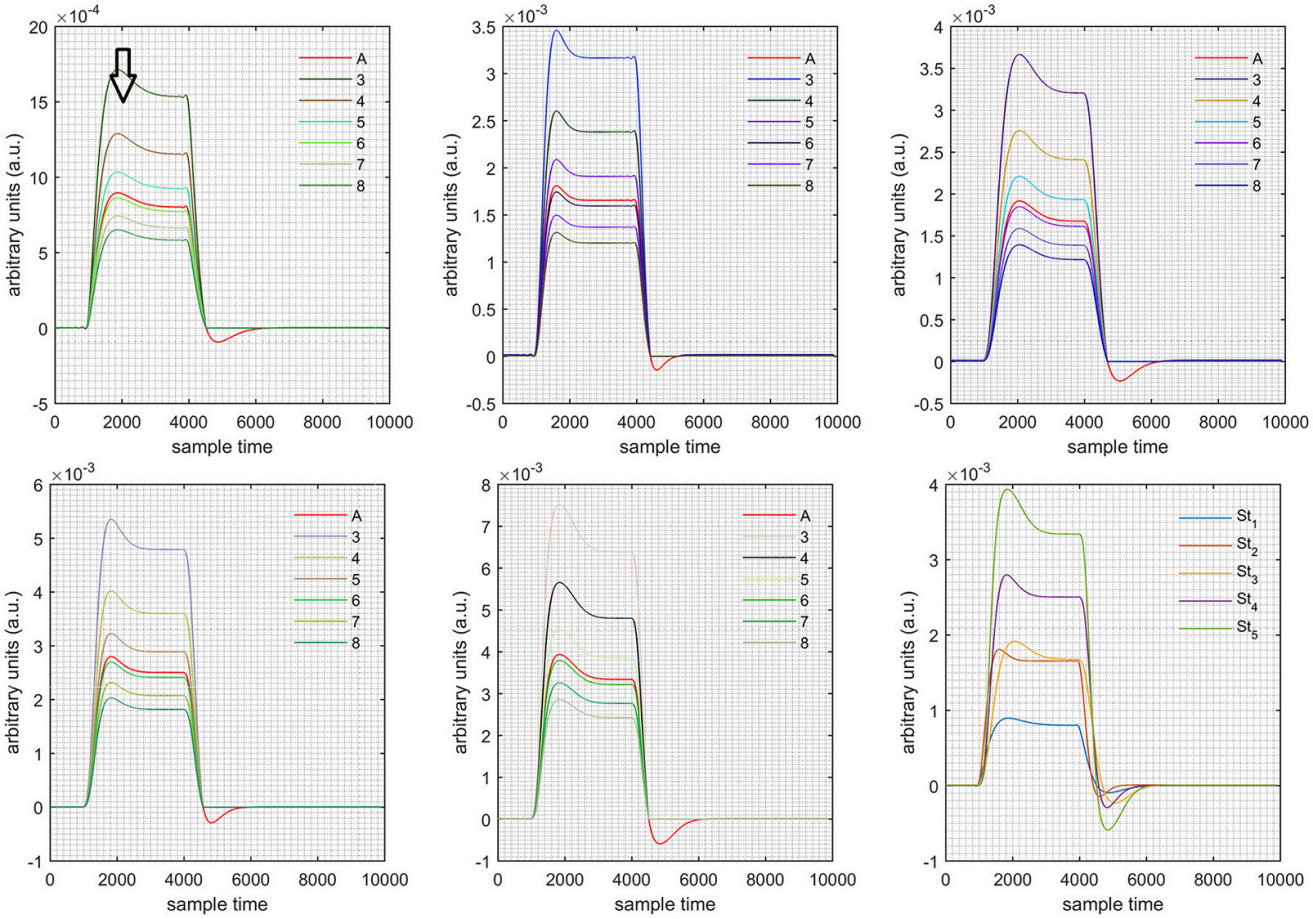
Results for first negative and second positive cases (Case: 4) with variation in value of $DPF^{\lambda_{2}}$ (3–8) under stimulation St_1_-St_5_ (top left, top middle, top right, bottom left, and bottom middle).

**Figure 12:**
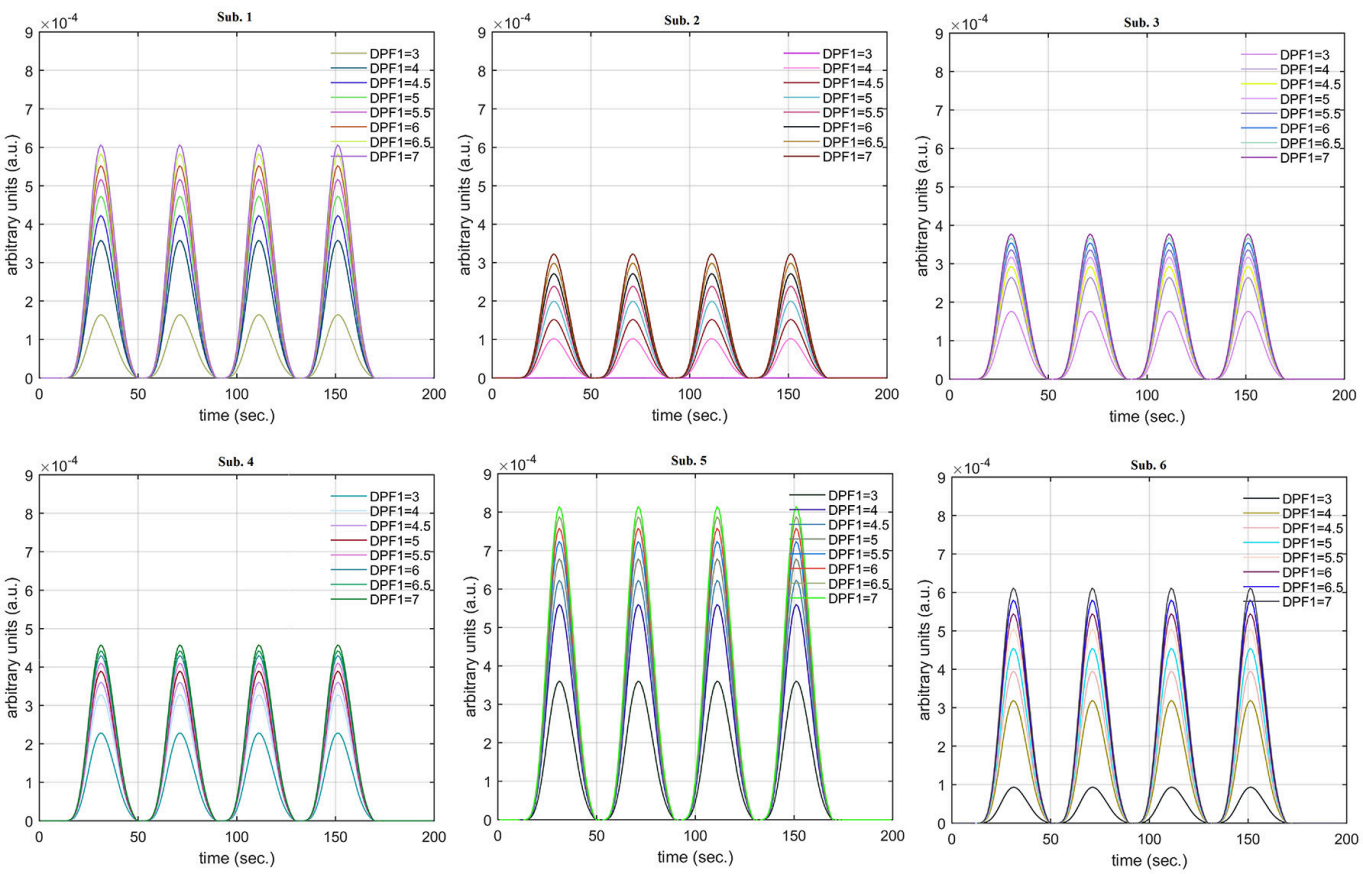
Results for fNIRS data set with variation in $DPF^{\lambda_{1}}$.

**Figure 13:**
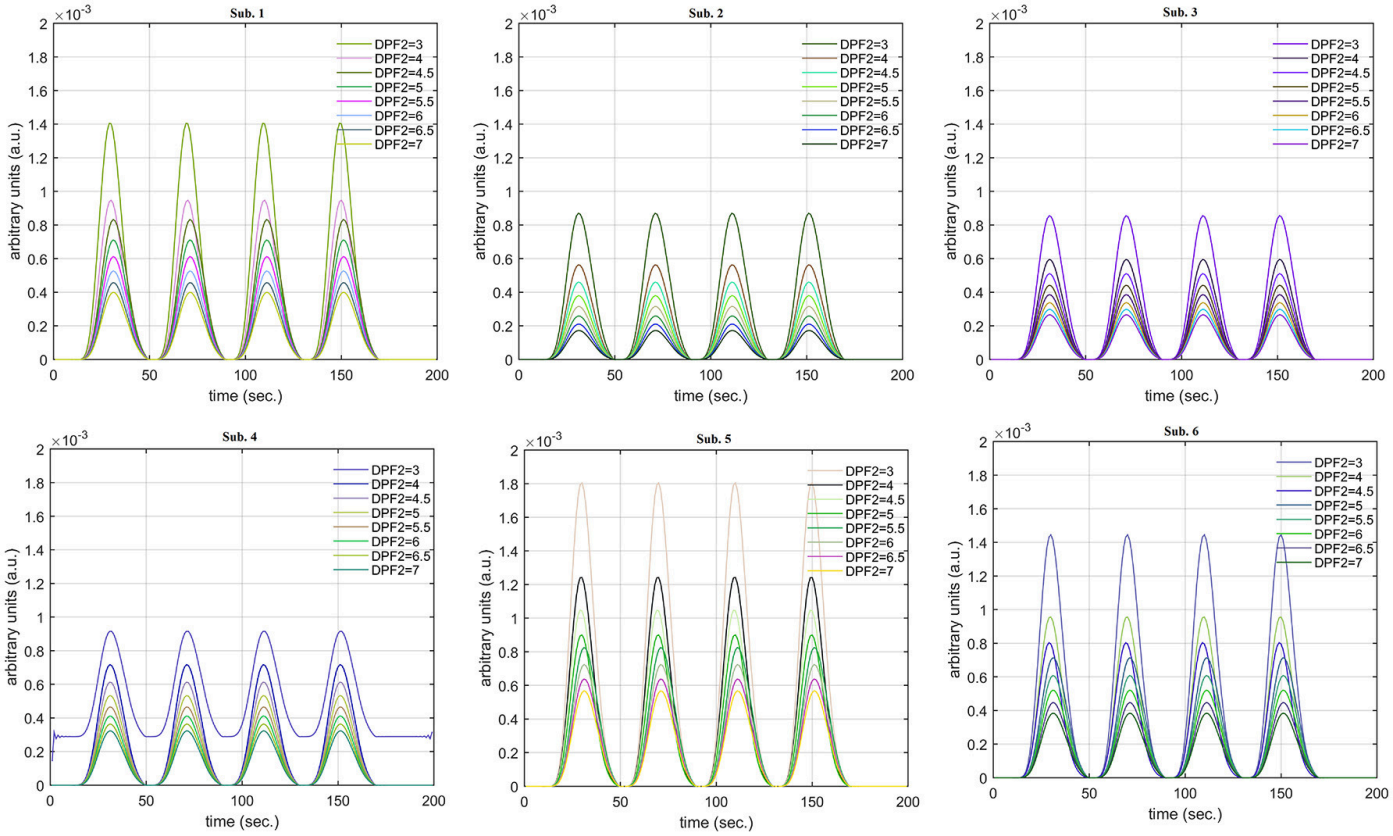
Results for fNIRS data set with variation in $DPF^{\lambda_{2}}$.

**Figure 14:**
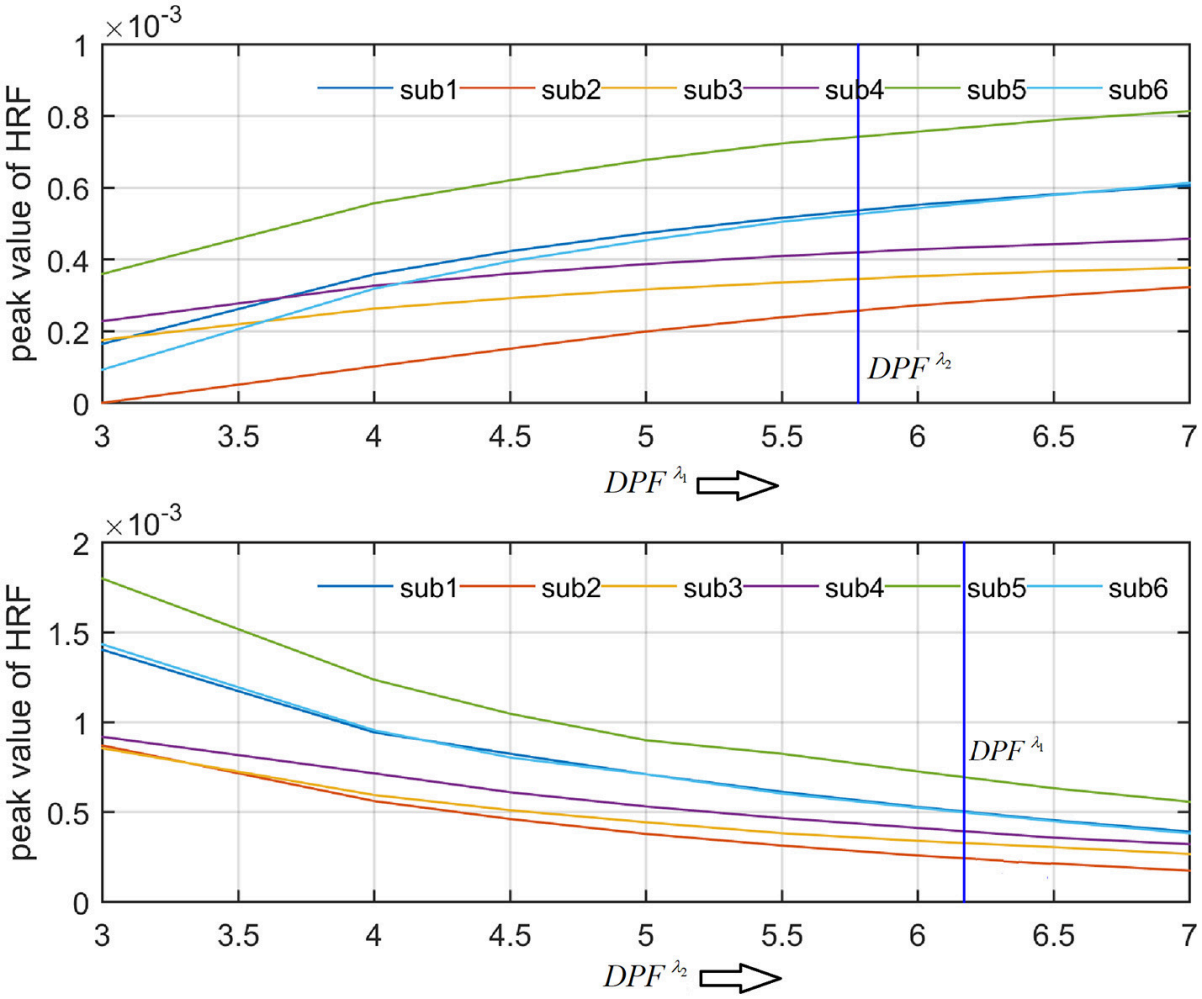
Effects of variations in $DPF^{\lambda_{1}}$ and $DPF^{\lambda_{2}}$ on peak of HRF for all subjects.

A schematic of the algorithm is shown in Figure 1. It displays the general idea of the study. Each time different set of values of DPF were used to analyze the output of neuro-activation optimization model and results were compared to comprehensively analyze the effects. The details of the experimental paradigm and source-detector separation and localization are shown in Figure 2. Sixteen set of source-detector pairs were utilized to measure motor cortex. The channel 3 is located at C3 location in 10–20 system. Figure 3 plots the simulated HRFs and their corresponding cHRFs for different task sessions. Figures 4–7 plot the effects of the variation of $DPF^{\lambda_{1}}$ on the HRFs corresponding to different stimuli (St_1_-St_5_). Similarly, Figures 8–11 show the effects of variations of $DPF^{\lambda_{2}}$ on HRFs corresponding to different stimuli (St_1_-St_5_). The *x*-axis in Figures 4–11 shows the sample time as mentioned before that the data is resampled at 100 Hz. Thus, each step of sample time corresponds to 0.01 s. It is obvious to analyze from Figures 4–11 that DPF has strong relation with the shape of HRF. A change in DPF value can affect the peak value of the main response as well as the depth of post-stimulus undershoot. In some cases DPF has no effect on main response (Figures 6, 10) and in some cases it has nothing to do with post-stimulus undershoot (Figures 7, 11). The reason behind this is that positive output is not possible in case: 3 (See section Discussion) and negative output is not possible in case: 4 (See section Discussion). Figure 12 summarizes the results for the six healthy subjects with respect to the effects of $DPF^{\lambda_{1}}$ change and DPF1 in the label represents the $DPF^{\lambda_{1}}$. Figure 13 plots the effects of $DPF^{\lambda_{2}}$ variation on real data sets and DPF2 in the label represents the $DPF^{\lambda_{2}}$. Figure 14 projects the effects of changes in the peak values of HRF as $DPF^{\lambda_{1}}$ (top plot) and $DPF^{\lambda_{2}}$ (bottom plot) are varied. The concentration change of HbO is calculated by using $DPF^{\lambda_{1}}$ and $DPF^{\lambda_{2}}$ from Equation (9). Figure 14 top plot displays the effects of variation of $DPF^{\lambda_{1}}$ keeping $DPF^{\lambda_{2}}$ at fixed value. The value of $DPF^{\lambda_{2}}$ is displayed by dark blue vertical line. Similarly, the bottom plot shows the value of $DPF^{\lambda_{1}}$ by vertical blue line while displaying the variation of $DPF^{\lambda_{2}}$ for all subjects.

## Discussion

fNIRS is an emerging neuro-imaging methodology that can be used in medical as well as BCI applications (Kamran et al., 2016). The observed time-series of optical data includes unnecessary (unrelated to cortical activity) information. This information is noise that renders extraction of the neuron-related information on a particular task more challenging. Noise is of different types: physiological noise, motion artifacts, systematic noise, and others. There also exist other factors operative in the process of converting fNIRS' observed optical densities, those can affect signal shape. The DPF is the important parameter in the conversion step, as it can affect signal attributes (Jasdzewski et al., 2003). The DPF is included to cover the actual distance traveled by light in the signal acquisition process (Maikala, 2010; Kamran et al., 2016). Several studies have reported the effect of the DPF on fNIRS signals (Lackowicz and Berndt, 1990; Duncan et al., 1993, 1995; Kohl et al., 1998; Jasdzewski et al., 2003; Schroeter et al., 2003, 2004; Talukdar et al., 2013). Hiraoka et al. (1993) reported that DPF has only valid for a homogenous medium but real human brain and head tissues have different optical properties. Thus, accurate quantification of NIRS signal is possible with information regarding nature of light transport through in-homogenous medium. Chatterjee et al. (2015) analyzed a Monte-Carlo based computational model within a single layer of tissue like human brain. Their finding concluded that optical path changes by changing the source-detector separation. The brain is highly scatter medium of NIR light. Thus, variation in path-length traveled by light photons is also possible. DPF depends upon the extra path traveled by light photons. Schroeter et al. (2004) commented on a very important point that DPF can vary intra-individually between several pixels and none of commercially available optical imaging system measures DPF for each pixel. Therefore, it is necessary to analyze its effects on the signal. In the present study, the effects of DPF change were analyzed for both simulated data sets and the healthy subjects' real data sets. The simulated data sets were generated using the method described in Kamran et al. (2015). These data sets were subjected to Equation (14) to solve for optical densities under constraints. Four different cases were resolved with Equation (14), and the results were compared with actual data using Equation (15). Later, different values of $DPF^{\lambda_{1}}$ and $DPF^{\lambda_{2}}$ were used to analyze the effects.

**Case 1:** Both of the optical densities were constrained to be positive. In this case, as $DPF^{\lambda_{2}}$ was changed from the lower value of 3 to the higher value of 8, a translation was observed along the y-axis in the upward direction (Figure 4), but as $DPF^{\lambda_{1}}$ was increased more, the translation decreased. Additionally, the post-stimuli undershoot decreased as $DPF^{\lambda_{2}}$ increased. Similarly, a translation along the y-axis in the downward direction was observed for the variation of $DPF^{\lambda_{2}}$ (Figure 8). The change in $DPF^{\lambda_{2}}$ did not have much affect on the post-stimulus undershoot. **Case 2:** Both of the optical densities were constrained to be negative. This case showed a translation along the y-axis in the downward direction as $DPF^{\lambda_{1}}$ as varied. The translation step became small with proximity to the actual HRF (Figure 5). There is no effects on the post-stimulus undershoot. A translation also was observed along the y-axis in the upward direction as $DPF^{\lambda_{2}}$ as changed from lower to higher values (Figure 9). The post-stimulus undershoot decreased with increasing $DPF^{\lambda_{2}}$, and the translation became too small as value $DPF^{\lambda_{2}}$ is increased. **Case 3:** The optical density related to wavelength λ_1_ was constrained to be positive, and the one related to wavelength λ_2_ as negative. In this case, there was no effect on the positive part of the HRF (Figures 6, 10) for variation in either $DPF^{\lambda_{1}}$ or $DPF^{\lambda_{2}}$. The post-stimulus undershoot decreased as $DPF^{\lambda_{2}}$ increased, and the increment of decrease became small as the $DPF^{\lambda_{2}}$ values increased (Figure 10). Similarly, a slight change in post-stimulus undershoot was observed as $DPF^{\lambda_{1}}$ increased (Figure 6). **Case 4:** The optical density related to wavelength λ_1_ was constrained to be negative, and the one related to wavelength λ_2_ as positive. In this case, no effect was observed on post-stimulus undershoot for either $DPF^{\lambda_{1}}$ or $DPF^{\lambda_{2}}$ variation. One of the main reasons for this is that negative output was not possible in this case. It was observed that the peak of the response decreased as $DPF^{\lambda_{2}}$ increased and that there was a slight change in the peak of HRF as $DPF^{\lambda_{1}}$ increased (Figures 7, 11).

The real data on the healthy human subjects were collected using fNIRS in a visuo-motor experiment. The data was fed to Equation (16), and the best possible HRF was estimated using cost function *J*_2_. The results (Figure 12) indicated that the peak of HRF increased as $DPF^{\lambda_{1}}$ was increased from lower to higher values. Also, there was an increment in the full width at half maximum (FWHM) of the HRF. Similarly, in contrast to the $DPF^{\lambda_{1}}$ variation, a decrement in the peak of HRF was observed as $DPF^{\lambda_{2}}$ was increased, resulting also in a decrement of FWHM (Figure 13). The changes in the peak values of HRF with variation of $DPF^{\lambda_{1}}$ and $DPF^{\lambda_{2}}$ are plotted in Figure 14. The results clearly show that a change in DPF corresponding to any wavelength significantly effects HRF shape. It is well known fact that in most BCI-fNIRS applications, features are observed from HRF attributes. Thus, a slight change in HRF on the basis of the DPF can lead to incurred error, misleading results, and reduced accuracy.

It was evident from the diffusion equation relating to the transportation of light through the homogeneous semi-infinite medium that the DPF depends on the reduced scattering coefficient, the absorption coefficient, and the source-detector separation (Scholkmann and Wolf, 2013). The human brain tissue causes high scattering of NIR light. The difference in structure and behavior of tissue is also possible among different subjects. Therefore, each human being respond differently to NIR light. That is due to the variation in the optical properties of the path-length of light photons. It also causes the change in the traveled distance of light photons. Talukdar et al. (2013) concluded that multiple layer of human scalp, skull, and brain tissues can effect scattering and thus DPF, which leads to systematic errors. Thus, the variation of DPF among the subjects is obvious. Similarly, the MBLL can interpret the dependency of HRF on DPF (Maikala, 2010). Therefore, it is worthwhile to analyze the effects of DPF change on NIR light based on human brain signals, particularly given that different brain areas have different scattering and absorption properties according to a subject's specific physiology and condition. A possible means of analyzing the effect of DPF on HRF is to split the HRF into three sections, namely pre-stimuli, main response peak, and post-stimuli undershoot. Therefore, a generic overview of the effects of DPF on HRF was presented in this paper.

## Conclusion

In this study, the effects of the DPF were analyzed for fNIRS-observed data. The observed optical densities were utilized to formulate an iterative optimization problem for estimation of the best possible HRFs for different values of DPF related to wavelengths λ_1_ and λ_2_. Different simulated data sets were generated for a combination of different stimuli and activation times. The simulated HRF was fed into an optimization problem in order to split it into two different optical densities for further analyses. Later, different values of DPF were used to regenerate actual data and its effects. In addition to the simulation of data, the motor cortices of six healthy human subjects were scanned for a DPF analysis of measured fNIRS signals. It was concluded that the DPF affects the attributes of the HRF and that correct values of DPF are required for accurate results and their proper interpretation.

## Author contributions

MAK has proposed the methodology for analysis, did simulations and wrote the first draft of the manuscript. MM has done literature survey, implementation of methodology, did simulations and helped in editing of manuscript before submission. MJ has supervised the research.

### Conflict of interest statement

The authors declare that the research was conducted in the absence of any commercial or financial relationships that could be construed as a potential conflict of interest.
